# Goal attainment scaling with older people in general practice: A feasibility study

**DOI:** 10.1016/j.ijnsa.2020.100015

**Published:** 2020-12-15

**Authors:** Sophie CE van Blijswijk, Jacobijn Gussekloo, Florentine M Heijmans, Annet W Wind, Wendy PJ den Elzen, Jeanet W Blom

**Affiliations:** a Department of Public Health and Primary Care, Leiden University Medical Center, Hippocratespad 21, Postzone V0-P, Postbus 9600, 2300 RC Leiden, the Netherlands; b Department of Internal Medicine, section Gerontology and Geriatrics, Leiden University Medical Center, Hippocratespad 21, Postzone V0-P, Postbus 9600, 2300 RC Leiden, the Netherlands; c Department of Clinical Chemistry and Laboratory Medicine, Leiden University Medical Center, Hippocratespad 21, Postzone V0-P, Postbus 9600, 2300 RC Leiden, the Netherlands

**Keywords:** Care-action-plan, Community-dwelling older people, Evaluation method, General practice, Goal attainment scaling, Goal setting, Individualised care, Personalised care, Practice nurses, Primary care

## Abstract

**Background:**

The impact of health problems on daily life and consequent treatment goals differ from person to person, particularly for older people with multiple health problems. Personalized care in general practice can help address these health problems, but evaluation of its effects remains difficult. In rehabilitation, a common approach to the evaluation of personalized care is Goal Attainment Scaling. This feasibility study assesses whether goal attainment scaling can also be applied to the evaluation of personal care for community-dwelling older people in general practice.

**Methods:**

General practices were invited to participate in this longitudinal, observational feasibility study. Practice nurses and general practitioners received training in care plans and goal attainment scaling. They were each asked to create care plans and goal attainment scales for patients (aged ≥75 years) and to carry out evaluations at three and six months. Professionals and patients both completed a short questionnaire to evaluate their experiences regarding the (dis)advantages of goal attainment scaling.

**Results:**

Professionals (*n*=10) and patients (*n*=23) were able to set goals and scales (*n*=57) for problems across five health domains (somatic, functional, social, psychological and communicative), but experienced difficulties formulating goals and corresponding goal attainment scaling levels. Reported benefits of goal attainment scaling were 1) important problems were addressed, 2) patients were involved and motivated to attain goals, and 3) evaluation was straightforward once a scale was created. Disadvantages were 1) difficult for older people, 2) time-consuming and complex for clinical practice.

**Conclusions:**

Goal attainment scaling shows potential benefit for clinical practice and general practice research in terms of the setting and evaluation of goals for community-dwelling older persons. Further research is needed to develop more standardized and less time-consuming goal attainment scaling methods.

What is already known?

• Community-dwelling older people would benefit from personalised care in general practices
• In the Netherlands, practice nurses often provide this care via individual care-action-plans
• Goal attainment scaling is used as an evaluation method for personalised care in other fields

What this paper adds?

• Use of goal attainment scaling in the evaluation of care-action-plans for community-dwelling older persons in general practices has important advantages, especially regarding the motivation of patients and evaluation of set goals
• Despite several attempts to reduce the necessary time investment, the time-consuming format still represents an obstacle to its implementation in daily practice

## Background

Older people frequently experience a multitude and diversity of health problems that cause (functional) limitations (Tinetti, 1986; van Houwelingen et al., 2015; den Elzen et al., 2009). Since the impact of these limitations varies from person to person, the priorities and goals of treatment also differ for each older individual (De Maeseneer and Boeckxstaens, 2012; Robben et al., 2011; Huijg et al., 2017). This suggests that care for older people in general practice should be personalized in terms of identification of individual health problems and attainment of personal goals (De Maeseneer and Boeckxstaens, 2012; Robben et al., 2011; Huijg et al., 2017). In many general practices in the Netherlands, a practice nurse (PN) trained in care for older people already works with healthcare plans in which a patient's personal problems and goals are recorded and prioritized. Advantages of these care plans are: 1) involvement of the older person in the setting of goals, 2) a transparent method to collect and prioritize all goals, and 3) awareness of the patients’ goals among healthcare professionals involved.

The next challenge is the efficient assessment of personal care aimed at attaining individual goals. In scientific research involving older people, standard questionnaires do not always fully capture the impact of an intervention on the diverse individual problems experienced by older persons (Smith et al., 2012; Blom et al., 2016; Rockwood et al., 1993; Yip et al., 1998). To successfully evaluate individual goal-oriented care, outcomes should encompass personal differences in goals and interventions (Steenbeek et al., 2011; Rockwood et al., 2003). In general practices, the diversity of patients, diseases and personal goals makes it important to be able to evaluate the attainment of all relevant goals per patient. One solution might be to use individual measurement tools (Blom et al., 2016; Mullis et al., 2011; Wright, 1996) such as Goal Attainment Scaling (GAS). GAS was designed for use in mental health and rehabilitation care (Steenbeek et al., 2011; Rockwood et al., 2006; Kiresuk and Sherman, 1968) and can measure individual progress towards (and beyond) individual goals. In short, a goal attainment scale is an individualised scale to evaluate whether a personal goal was or was not attained. To our knowledge, it has not yet been used to evaluate healthcare plans for community-dwelling older people in general practice. The aim of the present study was therefore to assess whether GAS is fit for purpose in the assessment of 1) the needs of older people in general practice, and 2) differences in scores over time at both the individual and group level.

## Methods

### Study design and population

This observational feasibility study had a longitudinal design and was conducted in general practices in Leiden and surrounding area, the Netherlands. Invitations to participate in this study were sent to general practitioners (GPs) who participated in an earlier trial on care plans for older people (Blom et al., 2016) and to GPs who act as trainers in general practice postgraduate training. GPs from the Leiden Network for Research in Primary Care were informed of the possibility of participating in the study via a newsletter, not a personal invitation. PNs did not receive personal invitations, but could be invited by their GP. The aim was to include ten general practices, with five patients per practice. The GPs and PNs (hereafter referred to as the ‘professionals’) selected older community-dwelling patients in accordance with the inclusion criteria (Table 1). The professional invited and interviewed the selected patients in order to compose a care plan in a manner that best fitted usual care in their practice (i.e. at the general practice or at home). Development of a care plan is increasingly becoming usual care in many general practices in the Netherlands. Additions needed for this study included a goal attainment scale and an evaluation using this scale. Information about the study was provided to patients by the professional and during the study the professionals continued to provide care as usual. Patients were informed that a decision to(not) participate in this study would not effect their relationship with their health professional or their usual care.

**Table 1:** Inclusion criteria.

aged ≥75 years
life expectancy ≥6 months (according to the professional)
living at home or in a retirement home
enlisted in a general practice
considered eligible (cognitively, mentally and physically capable) to compose a care plan according to the professional

### Care plans and goal attainment scaling

**Fig. 1:**
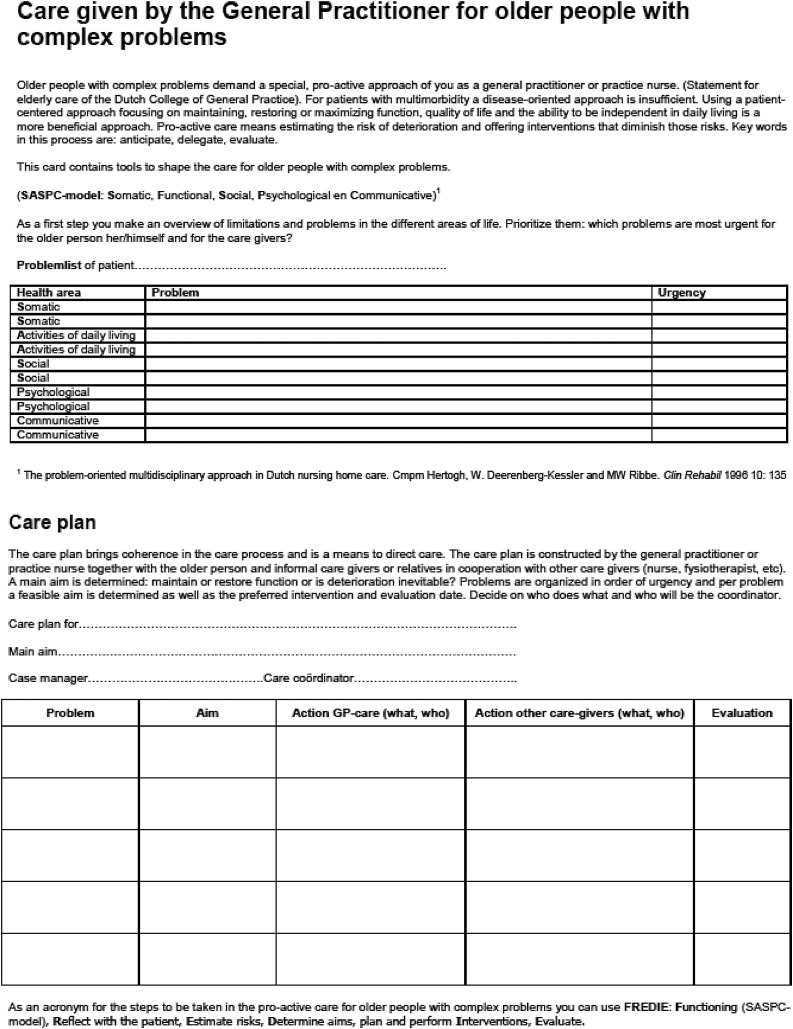
Format care plan from the Dutch College of General Practice and National Advisory committee for Primary Healthcare for Older people.

The aim of the care plan (Fig. 1) is to provide an overview of problems and actions relevant to the patient, to informal caregivers and to professional care providers. Problems are grouped per health domain and include somatic, activities of daily living, social, psychological and communicative domains. The care plan is jointly prepared by the professional, the patient and, if appropriate, the informal caregiver. Goals and priorities of the patient give direction to the care plan (Wind and Gussekloo, 2015). After identification of problems, those that are most urgent for the patient and the (informal) caregivers are prioritized. Achievable goals are then formulated for each problem and appropriate actions and an evaluation date are set. Actions related to GP care are described in column 3, while actions for the patient and for informal caregivers are described in column 4. Preparing a care plan and setting goals together with the patient can help structure goal-oriented care and stimulate patients to achieve these goals (Blom et al., 2016; Wind and Gussekloo, 2015; Stolee et al., 2012).

**Fig. 2:**
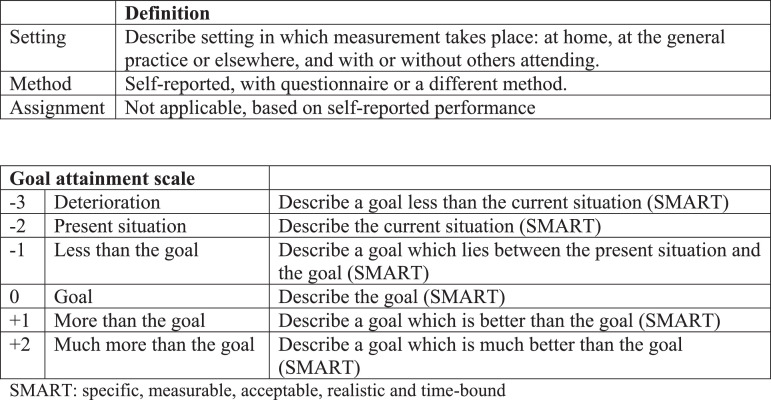
Format of six-point Goal Attainment Scale used in this study (based on Dekkers et al. (2011)).

Each professional was asked to compose care plans with their patients for at least two problems per patient. For the purposes of this study they were further asked to create a goal attainment scale for these problems together with their patients. The goal attainment scale (scale) was first introduced in 1968 and has been further developed since (Kiresuk and Sherman, 1968; Turner-Stokes and Williams, 2010). Steenbeek et al. (2011, 2005, 2008), Dekkers et al. (2011) adapted the original 5-point scale to include scores from −3 to +2 in order to allow visualization of deterioration, with −2 defined as the status at baseline and 0 as the formulated goal in this model. If a patient is making progress but has not yet attained a goal, a score of −1 can be assigned, with +1 (and +2) reserved for when a patient has made progress, even well beyond the goal (Fig. 2) (Steenbeek et al., 2011, 2005, 2008; Dekkers et al., 2011). This 6-point scale was chosen for this study since it allows visualization of goal attainment, of deterioration from baseline, as well as improvement without actual attainment of the goal. Furthermore, professionals were given the option to define current status as the goal (0) for those patients in whom they, in agreement with the patient, aimed to maintain current function rather than expect improvement.

In terms of scale development it is important that i) the patient (and caregiver) agree with the formulated goals and scale, ii) goals represent activities in which the patient participates, iii) all scale levels are specific, measurable, acceptable, realistic and time-bound (SMART), and iv) one variable of change is used per scale (Steenbeek et al., 2011, 2005; Dekkers et al., 2011). Self-reported performance was used to evaluate baseline status and attainment of the goal (Holsbeeke et al., 2009).

### Evaluation of care plans with GAS

Professionals were asked to evaluate each goal based on GAS after three and six months, using self-reported performance. Again, this could be implemented in a way that best fitted their usual care. Any additional comments (i.e. side-effects or reasons why a goal was not attained) could also be reported.

### Preparation: training and database with goals

**Fig. 3:**
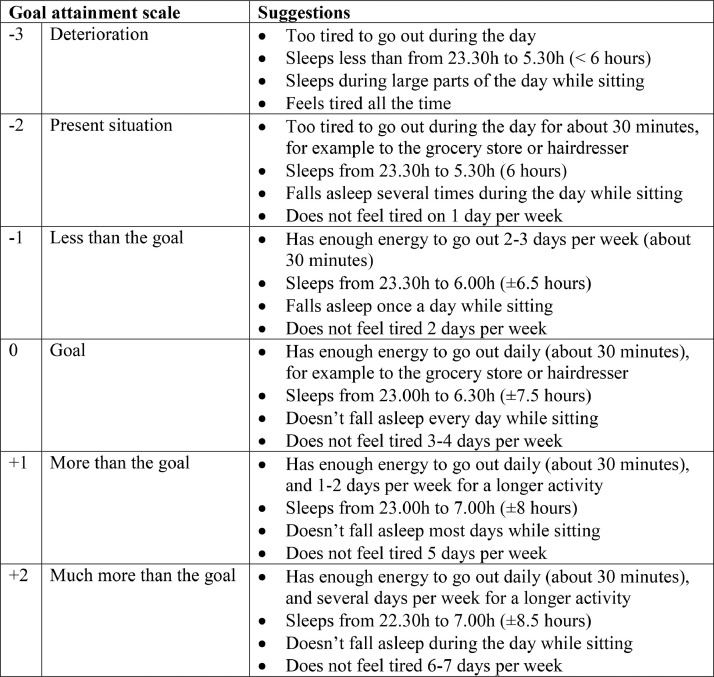
Translated examples from the ‘goal bank’ for tiredness/weakness.

Participating professionals received two 2-hour training sessions on evaluating care plans using GAS. Furthermore, examples of goals and scales were provided in a ‘goal bank’ based on complaints that hinder older people in daily life. These examples were either reported by older people (van Blijswijk et al., 2015) or were the most frequently mentioned problems in care plans (Blom et al., 2016; van Blijswijk et al., 2015). Translated examples from the ‘goal bank’ are given in Fig. 3. Three authors (SCEB, JWB and AW), together with senior GPs participating in the postgraduate training of GPs with a special interest in geriatric medicine, prepared the examples of goals and scales for these problems.

### Additional questionnaires

Professionals and patients were asked to complete a short questionnaire at baseline and at three and six months on their experience of working with GAS. The questions were developed by the research team based on the research questions. Professionals and patients were then asked to identify the advantages of working with GAS, together with the limitations experienced.

Patients’ age and sex, and the profession of the participating professionals, were reported. Professionals were asked to indicate if the participants’ health problems were complex, if the participants had frequent contact with their GP (>5 per year), if polypharmacy was an issue (>4 drugs per day), and if there were any other problems in daily life (i.e. cognitive, functional, visual or hearing problems, and/or incontinence).

All questionnaires were provided to the professionals at the end of the training sessions. Professionals joined a meeting at the end of the study to evaluate working with GAS.

### Data analysis

In summary, data available for analysis collected per patient included the completed care plan and GAS (baseline), evaluation with GAS at three and six months, questionnaires completed by older adults and professionals at baseline, at three and six months, together with notes from the meeting with professionals at termination of the study.

A study flowchart and the characteristics of both professionals and patients are described. Further analysis of quality of goals and scales, sensitivity to change and aspects of feasibility are described in the following paragraphs.

#### Quality of goals and scales

The type of problems reported in the care plans was described. In addition, the quality of the goals and scales were evaluated by a researcher (SCEB) and a medical student (FMH) and differences were discussed. Aspects of quality were:

(1) SMART formulation of the goals. Goals were assessed for specificity, measurability and time-boundness. Establishing whether a goal is acceptable and realistic is not possible without background knowledge of the patient.
(2) Change in only one variable per scale (i.e. if the aim is to extend the number of meters a person can walk, the assistance required should remain stable).
(3) If the agreed actions match the described problem and contribute to the attainment of the goal (Dekkers et al., 2011; Krasny-Pacini et al., 2016).

#### Sensitivity to change

To evaluate whether GAS can detect differences in scores over time for individual patients and at a group level, the raw score on the scale per individual problem at six months post-baseline is presented. The number of goals attained at three and six months post-baseline is presented as a percentage, as are the numbers of attained care plans.

#### Aspects of feasibility

The (dis)advantages experienced by professionals working with GAS (e.g. time spent on developing and evaluating a care plan) and the required training on care plans and GAS were both evaluated. The questionnaire answers, together with shared experiences and suggestions from the final study meeting, were listed, compared and summarized by two authors (FMH and SCEB). Patient opinions on working with GAS were analysed based on the questionnaires and their comments at baseline and during evaluations at three and six months. In view of the age of the population, some loss to follow-up was anticipated and the causes of loss to follow-up were examined.

### Ethical approval and informed consent

The Medical Ethical Committee of Leiden University Medical Center approved this study. All participating patients provided signed informed consent forms which were kept at the general practices.

## Results

**Fig. 4:**
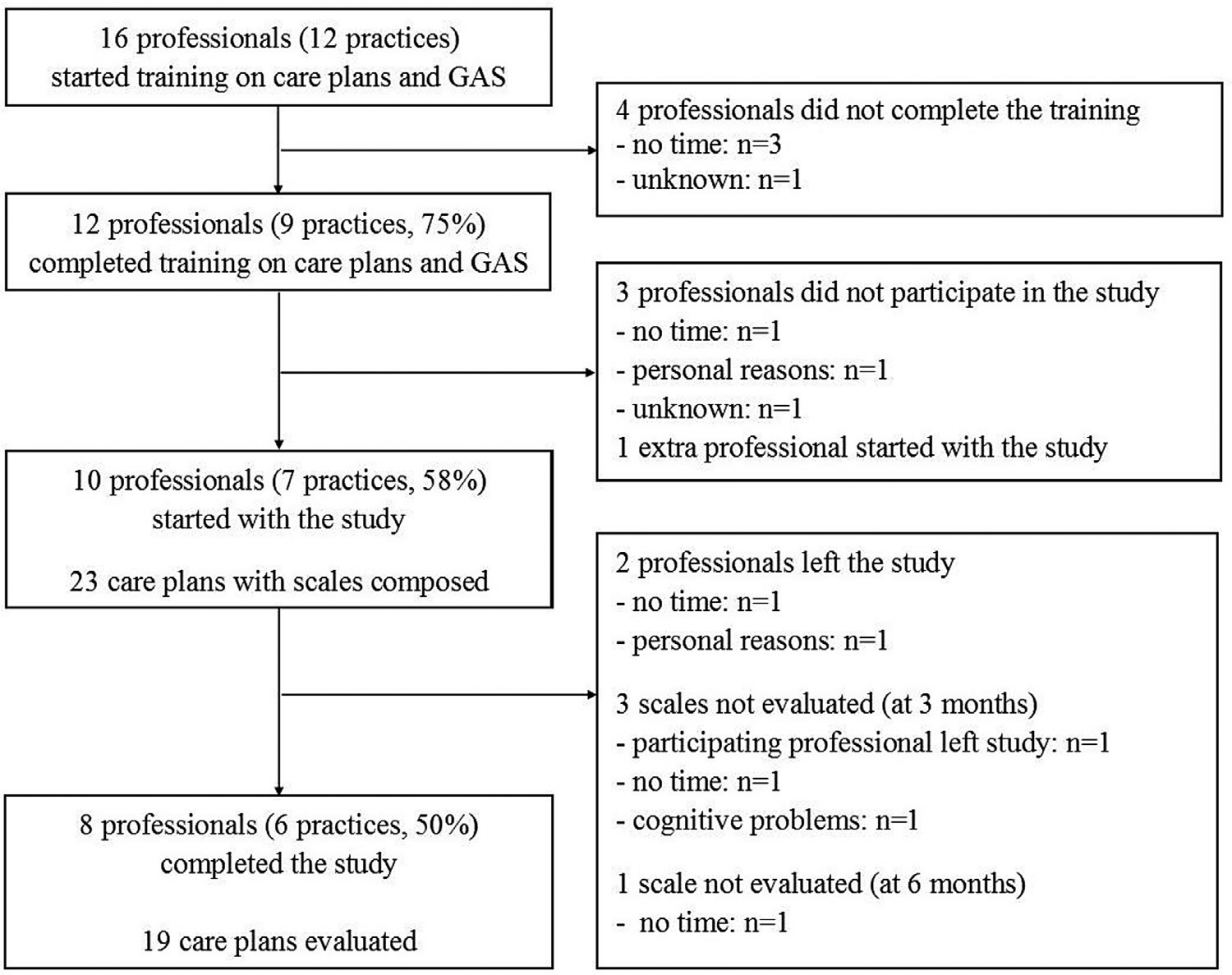
Flowchart of the study.

Of the 16 professionals (12 practices) who initially attended the two-session training on care plans and GAS, 10 (8 PNs and 2 GPs in 7 practices) eventually participated in the study (Fig. 4). All participating professionals had clinical experience in working with community-dwelling older people in general practice, but had no experience of working with GAS or other personal goal measurement instruments. In total, 23 care plans with scales (Fig. 4) for community-dwelling older people (median age 86, IQR 82–91 years; 91% female) were prepared. In the accompanying questionnaires (*n*=21) the professionals noted that 19 patients had complex health problems. Other characteristics of the patients were reported in table 2.

**Table 2:** Baseline characteristics of the participants (*n* = 23).

**Characteristic**	**n**	**%**
Median age in years (IQR; range)	86 (82–91; 73–98)	
Female	21	91
Patient with complex problems*	19	91
Patients with >5 contacts with GP per year*	9	43
Polypharmacy*	12	57
Cognitive problems*	3	14
Functional problems*	17	81
Problems with hearing/visual function*	9	43
Incontinence (urine/feces)*	6	29

⁎ 2 missing: *n* = 21.

### Quality of goals and scales

The total number of goals was 57; of these, most were either somatic (*n*=19) (e.g. pain, urinary incontinence) or functional (*n*=18) (e.g. problems with walking, visual limitations). Examples of the various goals illustrate the personal aspect of the care plans (Table 3).

**Table 3:** Examples of goals for patients with problems with walking and loneliness.

**Goals for problems with walking (*n*=9)**
Can walk to the city center and back without becoming tired
Time-up-and-go <12 s
Improve walking skills to >19 on the Performance-Oriented Mobility Assessment (POMA)
Can walk to end of the waterway with walker (500 m)
Can walk to her friend's
Walks with walker to bakery to do the shopping
Can walk in her own house without walker
Can walk around house for the elderly with walker
Is capable of walking longer distances allowing her to walk to the city center or library
**Goals for loneliness (*n*=8)**
Participates in an activity with a volunteer once a week
Goes to one or two activities a week and visits neighbours once a week
Has one out-of-house activity per day
Participates once per month in an activity with children/friends outside own living area
Visits city center with husband once a week
Joins the craft club in the nearby home for the elderly once every week
Keeps social contacts with friends and family at the current level
Joins an activity in the community center twice a week

At baseline, consolidation of current status (and no improvement) was the goal for five reported health problems in the care plans. Forty-six goals (81%) were considered to be ‘SMART’, 11 scales (19%) were incomplete and 2 goals (4%) did not match the problem. For 17 goals (30%) more than one variable was changed in the same scale.

### Sensitivity to change

Twenty care plans were evaluated at three months (Fig. 4) and all goals in the care plans could be evaluated. Of the 47 evaluated goals, 24 (51%) were attained or had improved beyond the goal (score 0, +1 or +2). At six months, 19 care plans were evaluated and of the 44 evaluated goals, 26 (59%) were attained, of which 12 had improved beyond the goal (score +1 or +2).

In addition, improvement without goal attainment was reported for 7 goals (16%), 10 goals (23%) remained at the same level without goal attainment, and for one goal (2%) the level deteriorated (Table 4).

**Table 4:** Description of goals and evaluation.

**Variables**	**n**	**%**
**Goals at baseline per person (*n*=23)**		
1 goal	3	13.0
2 goals	12	52.2
3 goals	5	21.7
4 goals	2	8.7
6 goals	1	4.3
Median minutes worked on scales (range)*	30 (15–180)	
**Evaluation at 3 months (*n*=20)**		
Attainment (score 0, +1 or +2) of goals (*n*=47)	24	51.1
Median minutes worked on evaluation (range)	30 (10–60)	
**Evaluation at 6 months (*n*=19)**		
Attainment score of goals (*n*=44)		
−3 – deteriorated	1	2.3
−2 – remained at the same level	10	22.7
−1 – improved, but goal not attained	7	15.9
0 – goal attained	14	31.8
+1 – a little better than goal	5	11.4
+2 – much better than goal	7	15.9
Attained goals per health domain (*n*=44)		
Somatic (*n*=16)	7/16	43.8
Functional (*n*=14)	8/14	57.1
Social (*n*=5)	3/5	60.0
Psychological (*n*=8)	8/8	100
Communicative (*n*=1)	0/1	0.0
Median minutes worked on evaluation (range)	20 (10–60)	

⁎ 4 missing, *n* = 19.

### Aspects of feasibility

For most patients, two goals (range 1–6) were set. However, whereas most goals were set by the PN together with the patient, the scales were often set later by the PN without patient input. On average, the additional time investment to compose scales was 30 min per patient (Table 4).

Three care plans were not evaluated at three and/or six months due to time constraints on the professional. One care plan was not evaluated as a result of cognitive problems developed by the older person (Fig. 4). Most care plans were evaluated by the PN together with the patient. At three months this evaluation required 30 min; at six months it took 20 min (Table 4).

### The advantages and disadvantages experienced

The 12 professionals who completed the two training sessions felt that they could work with the method in general practice. Upon request, a third session was planned to facilitate discussion of any difficulties encountered. Several factors relevant to patients and professionals were identified in the questionnaires and were brought up during this meeting. These are presented below as advantages or disadvantages.

Advantages:

  • Patient involvement: patients felt they had been ‘heard’ and that their sense of responsibility had been enhanced. The patients were motivated by the steps already attained. The majority of patients (*n*=16) agreed on the importance of described problems.
  • Overview for both patient and professional: patients and professionals were more aware of important problems. Formulating current status and scales helped professionals to visualize a realistic goal and possible steps towards it.
  • Easy evaluation: professionals stated that evaluations using GAS are quick, provide insight into a new situation, and aid decision-making regarding subsequent interventions. Of the 18 patients who completed the questionnaire at baseline, two found the evaluation of goal attainment difficult. Due to the structured description of the goal and scale, a professional could evaluate goals set by a colleague.

Disadvantages:

  • Difficult for older people: of the 18 patients who completed the questionnaire at baseline, four had difficulty describing their problems to professionals, and seven had difficulty describing the goals they wished to reach.
  • Too time-consuming and complex for use in clinical practice: professionals reported difficulty formulating SMART goals and various levels of attainment. The problems were sometimes intertwined and it felt unrealistic to separate them. Setting all levels of attainment felt artificial in clinical practice, the process was time-intensive, and unexpected developments in the personal life or health of patients sometimes had such a major impact that goals were suddenly no longer realistic or were less important.

Nevertheless, it was suggested that working with the method would become easier after gaining more experience, and the goal bank helped in formulating goals. Some professionals suggested working with goals without a scale, or with a shorter scale ranging from −1 to +1, in order to reduce the amount of time needed in clinical practice.

## Discussion

### Summary

Professionals and patients were able to use GAS in general practice in the care of older people with complex problems, and goals could be set for problems across all five health domains. Although setting goals was time-intensive and had drawbacks concerning direct implementation in general practice, evaluation of goals with GAS was easy and changes in the attainment of goals were well visualized using GAS.

### Comparison with literature

Many studies on goal setting and evaluation emerge from geriatric rehabilitation (Stolee et al., 2012; Steenbeek et al., 2005; Steenbeek et al., 2007; Stolee et al., 1999; Stolee et al., 1999; van Seben, Reichardt, Smorenburg, and Buurman, 2017). GAS is known to be a promising approach to the evaluation of personalized care in these more controlled settings, although the time needed to set SMART goals and scales is often cited as an major limitation (Steenbeek et al., 2008; Turner-Stokes et al., 2009; Grant and Ponsford, 2014). Health professionals found goal setting difficult and this was further complicated by the specific characteristics of older people in general practice (i.e. patients often have complex problems, and goals such as reducing loneliness are difficult to quantify) (Stolee et al., 1999; Siegert and Taylor, 2004; Parsons and Parsons, 2012). Furthermore, older people are less likely to take an active role in their own healthcare, although individual differences are large (Ekdahl et al., 2010; Arora and McHorney, 2000). All of these factors were encountered during the present study. However, despite these challenges, it was found that GAS can visualize changes over time in personal goals set for community-dwelling older people, a conclusion supported by other studies (Rockwood et al., 2003; Verdoorn et al., 2018).

GAS was originally a 5-point scale, but in previous research several different scales have been used in various settings (Rockwood et al., 2003; Turner-Stokes and Williams, 2010; van Seben, Reichardt, Smorenburg, and Buurman, 2017; Turner-Stokes et al., 2009; Turner-Stokes, 2009). A 6-point scale was used for the present study (Steenbeek et al., 2008), and despite differences to the 5-point scale, the 6-point scale retains the original goal attainment levels, allowing comparison to the outcomes of earlier studies based on the 5-point scale (Turner-Stokes and Williams, 2010; Steenbeek et al., 2005; Steenbeek et al., 2007). This possibility to compare may be valuable in future trials using a 5- or 6-point goal attainment scale as an outcome measurement.

Examples of other frequently used tools designed to measure the effect of personalized healthcare include the Canadian Occupational Performance Measure (COPM) (Carswell et al., 2004; Doig and Fleming, 2015), and the disease-specific Patient-Specific Index (PSI) (Wright and Young, 1997). The PSI has interesting properties when used to evaluate a specific disease; however, the present study aimed to evaluate a diverse range of complaints and limitations unrelated to any specific disease. COPM also shares this property, but a comparison between COPM and GAS showed that while COPM is more time-efficient (Cusick et al., 2006), GAS is more sensitive to change (van Seben, Reichardt, Smorenburg, and Buurman, 2017). In addition, GAS can be used to complement the care plans often composed in general practice for older people with complex problems (Genootschap, 2007). The inter-rater reliability was moderate to high in other studies (Hurn et al., 2006; Stolee et al., 1992). Rockwood et al. compared GAS to several alternative outcome measurements (e.g. the Barthel Index, etc.) and showed that GAS was the most suitable for showing changes in performance over time (Rockwood et al., 1993; Rockwood et al., 2003).

### Strengths and limitations

GAS could potentially be a valuable addition to usual care based on care plans. Important advantages included the identification of the most relevant problems according to the majority of patients, the setting of appropriate goals and actions, and the involvement and motivation to work on the attainment of their personal goals amongst patients. In this study, the aim was to interfere as little as possible with usual care, and professionals could therefore choose how they wanted to implement GAS in their practice. The choice of the 6-point scale proved a good match with clinical practice in which deterioration, maintenance of the status quo or small improvements are often seen.

The most important limitations of the present study were the small number of care plans developed and evaluated, and dropout amongst professionals during different stages of the study. Dropout was mainly due to the heavy work load and time constraints faced by PNs in general practice, combined with the time required by GAS. As previous studies had shown that GAS was considered time-intensive, mainly due to the need to compose the many scale levels (Yip et al., 1998; Stolee et al., 2012; Steenbeek et al., 2008), the aim was to reduce the time requirement as much as possible. All participating professionals were therefore offered two training sessions on GAS and access to a pre-prepared ‘goal bank’. Both were evaluated as positive and helpful, but professionals still felt that working with GAS was difficult and time-consuming. The setting of SMART goals and composing a scale posed especially difficult challenges. As a result, the number of care plans and scales received did not meet the number originally envisaged for the study. A further drawback was that, due to time constraints, most professionals composed the scales without input from the patient, since they expected this process would be too difficult for/with the patient. This approach may have resulted in steps that were unrealistic (e.g. too large/too small) for the patient, and less patient involvement than might have otherwise have been the case (Stolee et al., 2012; Rockwood et al., 1997).

### Suggestions for research

Although some major challenges still require attention, GAS is fit for purpose. Standardization of the process of problem identification and goal setting (e.g. structured interviews in which routines and problems during a regular day are discussed), evaluation, and the time required to work with GAS all require greater attention, without abandoning the specific features of GAS that make it suitable for the evaluation of personalised care (Yip et al., 1998; Turner-Stokes and Williams, 2010; Grant and Ponsford, 2014; Bovend'Eerdt et al., 2011). GAS can be influenced by professionals through the setting of too easily attainable goals, as seen in earlier studies. Although this might be desirable in clinical practice as a way to show patients that (small) steps are attainable, it can be problematic when GAS is used to evaluate the impact of an intervention. Possible influences on outcomes for other purposes (i.e. research, effectiveness studies) and how these can best be handled should be further investigated (Bovend'Eerdt et al., 2011; Steenbeek et al., 2010).

### Implications for clinical practice

GAS was found to be helpful when evaluating and, where necessary, adjusting care plans. Working with GAS in general practice has advantages, both as an outcome measurement (i.e. visualizing steps towards a goal and simplifying evaluation) and as an intervention with a potentially positive effect on care for community-dwelling older people (e.g. enhancing patient involvement, structuring healthcare when working with several professionals). To fully benefit from GAS, goal evaluation should be well-implemented (Grant and Ponsford, 2014; Turner-Stokes, 2009). Formulating SMART goals and appropriate actions together with the patient helps to achieve more insight into problems relevant for both patient and professional and enhances patient involvement (Grant and Ponsford, 2014; Verdoorn et al., 2018; Schulman-Green et al., 2006). It is worthwhile exploring whether patient participation in the setting of scale levels will further enhance their involvement (Stolee et al., 2012), as discussing the different steps of the scale with the patient could help in defining a specific goal and setting this goal realistically. To help professionals stimulate patients to think about their personal goals, it might be useful to place more focus during training sessions on communication around goal setting. In our opinion it is also worth exploring the benefits of sending a short questionnaire on current health problems and personal goals to the patient before their visit to compose a care plan. This will hopefully give patients more time to reflect on and formulate personal goals.

Further implementation of GAS in clinical practice will require support in order to enhance the quality of the goals and scales, and to reduce the amount of time involved. An interesting suggestion worth exploring further is to reduce the number of steps on the scale that professionals need to describe from six to three (Turner-Stokes, 2009) (see Fig. 2).

## Conclusions

Professionals and community-dwelling older patients can use GAS to set and evaluate goals in general practice, and changes in the attainment of goals are visible. However, the method remains time-intensive. In conclusion, GAS is fit for purpose in the evaluation of care in general practice for community-dwelling older people, although process standardization, further information on inter-rater reliability and a reduction in the amount of time required are all needed. Further research should focus on tackling these issues in order to ensure that they do not continue to detract from the benefits of GAS.

## Credit author statement

SCEB, JG, AWW, WPJE and JWB made substantial contributions to conception and design;

SCEB, FMH, AWW and JWB made substantial contributions to acquisition of data;

SCEB, JG, FMH, WPJE and JWB made substantial contributions to analysis and interpretation of data;

All authors have been involved in drafting the manuscript or revising it critically for important intellectual content;

All authors have given final approval of the version to be published.

All authors take public responsibility for appropriate portions of the content and agree to be accountable for all aspects of the work in ensuring that questions related to the accuracy or integrity of any part of the work are appropriately investigated and resolved.

## Declarations of Competing Interest

None.
